# Let Visuals Tell the Story: Medication Adherence in Patients with Type II Diabetes Captured by a Novel Ingestion Sensor Platform

**DOI:** 10.2196/mhealth.4292

**Published:** 2015-12-31

**Authors:** Sara H Browne, Yashar Behzadi, Gwen Littlewort

**Affiliations:** ^1^ University of California, San Diego School of Medicine La Jolla, CA United States; ^2^ Specialists in Global Health Encinitas, CA United States; ^3^ Proteus Digital Health Redwood City, CA United States; ^4^ University of California, San Diego Machine Perception Laboratory La Jolla, CA United States

**Keywords:** ingestion sensor platform, data visualization, time domain methods, medication adherence, patient self-management

## Abstract

**Background:**

Chronic diseases such as diabetes require high levels of medication adherence and patient self-management for optimal health outcomes. A novel sensing platform, Digital Health Feedback System (Proteus Digital Health, Redwood City, CA), can for the first time detect medication ingestion events and physiological measures simultaneously, using an edible sensor, personal monitor patch, and paired mobile device. The Digital Health Feedback System (DHFS) generates a large amount of data. Visual analytics of this rich dataset may provide insights into longitudinal patterns of medication adherence in the natural setting and potential relationships between medication adherence and physiological measures that were previously unknown.

**Objective:**

Our aim was to use modern methods of visual analytics to represent continuous and discrete data from the DHFS, plotting multiple different data types simultaneously to evaluate the potential of the DHFS to capture longitudinal patterns of medication-taking behavior and self-management in individual patients with type II diabetes.

**Methods:**

Visualizations were generated using time domain methods of oral metformin medication adherence and physiological data obtained by the DHFS use in 5 patients with type II diabetes over 37-42 days. The DHFS captured at-home metformin adherence, heart rate, activity, and sleep/rest. A mobile glucose monitor captured glucose testing and level (mg/dl). Algorithms were developed to analyze data over varying time periods: across the entire study, daily, and weekly. Following visualization analysis, correlations between sleep/rest and medication ingestion were calculated across all subjects.

**Results:**

A total of 197 subject days, encompassing 141,840 data events were analyzed. Individual continuous patch use varied between 87-98%. On average, the cohort took 78% (SD 12) of prescribed medication and took 77% (SD 26) within the prescribed ±2-hour time window. Average activity levels per subjects ranged from 4000-12,000 steps per day. The combination of activity level and heart rate indicated different levels of cardiovascular fitness between subjects. Visualizations over the entire study captured the longitudinal pattern of missed doses (the majority of which took place in the evening), the timing of ingestions in individual subjects, and the range of medication ingestion timing, which varied from 1.5-2.4 hours (Subject 3) to 11 hours (Subject 2). Individual morning self-management patterns over the study period were obtained by combining the times of waking, metformin ingestion, and glucose measurement. Visualizations combining multiple data streams over a 24-hour period captured patterns of broad daily events: when subjects rose in the morning, tested their blood glucose, took their medications, went to bed, hours of sleep/rest, and level of activity during the day. Visualizations identified highly consistent daily patterns in Subject 3, the most adherent participant. Erratic daily patterns including sleep/rest were demonstrated in Subject 2, the least adherent subject. Correlation between sleep /rest and medication ingestion in each individual subject was evaluated. Subjects 2 and 4 showed correlation between amount of sleep/rest over a 24-hour period and medication-taking the following day (Subject 2: *r*=.47, *P*<.02; Subject 4: *r*=.35, *P*<.05). With Subject 2, sleep/rest disruptions during the night were highly correlated (*r*=.47, *P*<.009) with missing doses the following day.

**Conclusions:**

Visualizations integrating medication ingestion and physiological data from the DHFS over varying time intervals captured detailed individual longitudinal patterns of medication adherence and self-management in the natural setting. Visualizing multiple data streams simultaneously, providing a data-rich representation, revealed information that would not have been shown by plotting data streams individually. Such analyses provided data far beyond traditional adherence summary statistics and may form the foundation of future personalized predictive interventions to drive longitudinal adherence and support optimal self-management in chronic diseases such as diabetes.

## Introduction

Medication adherence is defined as “the extent to which patients take medications as prescribed by their health care providers” [1]. An estimated 50% of patients do not adhere to prescribed medication regimens over time [1]. In the United States, US $100 billion in unnecessary hospitalizations and a total direct and indirect estimated cost of US $290 billion occurs from non-adherence annually; while non-adherence is estimated to cost the European Union €1.25 billion annually [1-4].

Any attempt to address this problem is complicated by the absence of reliable measurement of medication adherence, a difficulty that has plagued medicine since the days of Hippocrates [5]. The current gold standard is directly observed therapy, where a health care worker watches a patient swallow each dose of medication. This is expensive and impractical in chronic disease management. Alternatively, patient questioning, pill counts, and prescription refill rates have been used as methods for assessing adherence, but these have been shown to be inaccurate [1,6]. Electronic monitoring methods such as medication event monitoring systems, which rely on the opening of an electronic cap or patient dispensation of medication as a proxy measure of ingestion, emerged in the 1980s. The limitations of these systems, such as mismatches between electronic cap opening and actual intake, or patients obviating electronic assessment by decanting pills into a different container, have been well documented [7-10]. None of the methods of assessing medication adherence described above monitor actual medication ingestion, nor do they include any physiological measures such as activity, heart rate, or sleep/rest.

### A Novel Sensor Platform to Monitor Medication Adherence and Self-Management

A novel technology, Digital Health Feedback System (DHFS), is now available that allows for the date- and time-stamping of actual ingestions of oral medications rather than surrogate measures of ingestion. The system has been shown to be safe, reliable, and highly accurate, with a positive detection accuracy of sensor-detected ingestions of approximately 99.1% (321/324 ingested under direct observation; 95% CI 97.3-99.7) [11]. In the renal transplant population, the system accurately detected the ingestion of two sensor-enabled capsules taken at the same time with a detection rate of 99.3% (n=2376) [12]. The system is approved by the US Food and Drug Administration (FDA) and CE marked (European Conformity marking) (August 2012). It is the only system ever approved by the FDA for the measurement of medication adherence (July 2015).

**Figure 1:**
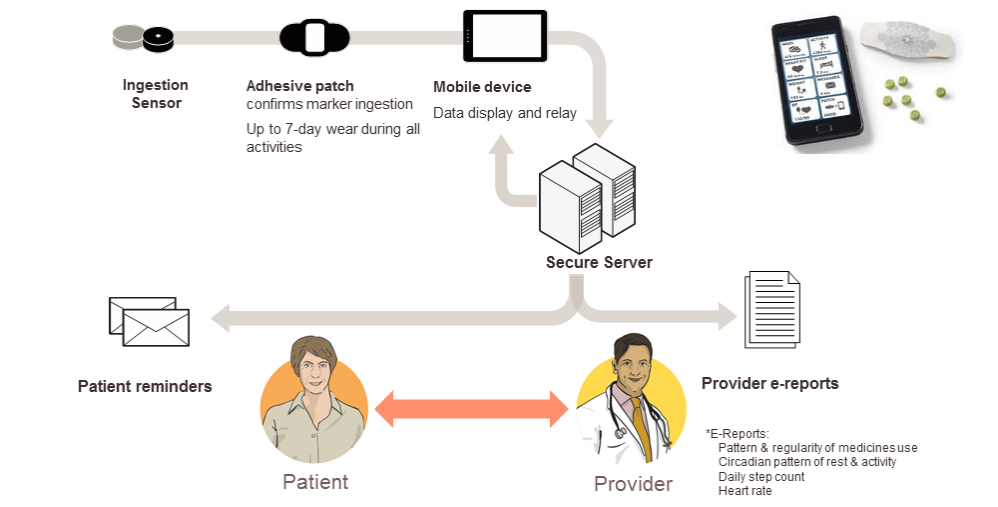
Overview of the Digital Health Feedback System to monitor medication adherence and self-management (figure courtesy of Proteus Digital Health).

This system consists of a 1 mm^3^ edible sensor (1 x 1 x.45 mm microchip), coated with very thin layers of commonly ingested excipients (ie, minerals and metals), and a small detector patch worn on the torso. When ingested with medication, the sensor readily separates from the carrier, is energized, and communicates with the adhesive-backed detector patch worn on the torso. The detector patch interprets the information as unique to the ingested sensor and records it along with physiological metrics including heart rate, step activity, and sleep/rest. All of the recorded data are sent wirelessly to a paired device, such as a mobile phone or a personal computer, and are subsequently uploaded to a secured, centralized data storage location (Figure 1) [12-15]. The patch can be configured to acquire various physiologic data at predetermined intervals. Individuals using the system generate dense physiological data that can be viewed in combination with the patient’s medication ingestion data.

### Continuous Wearable Sensor Platforms Require Novel Modes of Analysis

As described above, the DHFS includes a continuous wearable monitor patch and generates dense individual data on adherence and physiological measures. A patch, sampling every 5 minutes, can generate over 1000 data events per patient in a 24-hour period. In order to be interpretable and useful to a health care provider and patient, these dense data require novel methods of analysis not used previously in adherence monitoring. Currently, adherence data are traditionally reported as summary statistics, that is, “taking adherence,” the percentage of doses taken in relation to the total number of prescribed doses and “timing adherence,” the percentage of doses taken during a prescribed time interval. Such summary statistics derived from the ingestion sensor platform, while highly accurate in comparison to any other adherence assessment, will not reflect the richness of information that the entire DHFS system is capable of providing.

For dense DHFS data, we reasoned the use of more advanced analyses incorporating visualization methods were needed. Information visualization enhances human cognition in multiple ways, including enhancement of pattern recognition, such as when information is organized in space by its time relationships, and supporting the ease of perceptual inference of relationships that are otherwise more difficult to induce [16]. Thus, visualizations could capture dense data and, if well designed, may be able to quickly inform health care workers and be easily understood by patients with minimal training. The issue of how to present large amounts of continuous dense data to a patient and health care audience, and the study of effective ways of using visualizations to support the analysis of large amounts of medical data, is currently acknowledged as an area of critical need [17]. Visual analytics, defined as the science of analytical reasoning facilitated by advanced interactive visual interfaces [16], is an emerging discipline that has shown significant promise in addressing many of these information overload challenges [18]. One of the major figures in the field of visual analytics is Edward Tufte, who encourages the use of data-rich visualizations that present all available data without editing. He emphasizes that data-rich graphics can actually reveal what the data mean [19]. Furthermore, it is unclear how physiological measures can be used to increase understanding of individual patient medication ingestion patterns and self-management beyond traditional adherence summary statistics. Exploratory analyses using data-rich visualizations may indicate how this might be done. Thus, we explored combining multiple data streams of medication ingestion and physiological data together in novel data-rich visualizations to see if we could capture longitudinal patterns of medication-taking behavior and self-management in individual patients with type II diabetes. If successful, we reasoned that visualization data may provide information for individually tailored interventions that could support medication adherence.

The visualizations in this paper were derived from the data output of a trial of the DHFS system in patients with type II diabetes. Time domain methods were used to graph signal changes over time, visualizing multiple signals simultaneously. The trial included 5 patients each taking metformin twice daily over 37-42 days [13,20] and was a user experience study designed to evaluate the use of the technology. Thus, our work focuses on visualizations capturing longitudinal patterns of medication-taking and self-management over the course of the study; it does not focus on glucose control.

As an initial approach, we investigated whether varying the time interval over which data are analyzed and visualized provides different insights into patients’ medication-taking behavior. Thus separate analyses were performed looking at data for each individual over (1) the entire study period, (2) 24-hour periods, and (3) weekly time periods. Visualizations generated attempted to explore relationships that would be of value in supporting patient medication adherence and self-management in this chronic disease state.

## Methods

### User Experience Trial

Data analyzed were from a user experience study, which was a prospective, single-arm, observational cohort study (conducted by Diabolo Clinical Research) that aimed to characterize the at-home adherence patterns of 5 patients with type II diabetes to oral metformin [20]. The study captured each subject’s medication adherence and physiological data using the DHFS. Components of DHFS networked self-management system have been previously outlined in detail (see [20] and Figure 1). Subjects were instructed to continuously wear the personal monitor (RP2, Proteus Digital Health), co-ingest an edible sensor (IEM version DP4.2, Proteus Digital Health) whenever taking metformin, and to take one weight measurement daily (Medical Scale A&D) [13,20]. The subjects were also instructed to take at least one pre-meal fasting blood glucose measurement daily, preferably in the morning to provide a fasting glucose measurement, with a wireless glucometer (OneTouch Ultra2, Life Scan) and test strips [13,20]. Mobile phones used were Android 2 Global 2.2 operating system (Motorola). The data from the telemetered devices were then integrated into the DHFS system. Full ethical approval for the study was obtained from Ethical and Independent Review Services, Corte Madera. All participants signed an informed consent.

### Data Analysis and Visualization Generation Methods

We analyzed Microsoft Excel and Matlab files exported from the DHFS database and wireless glucose monitor, containing individual subject’s DHFS outputs of pulse measured every 10 minutes, activity recorded every 5 minutes, sleep/rest, and glucose.

Standard summary statistics in percentages were calculated across all subjects for (1) taking adherence, defined as the number of ingestible sensor tablet detections by the personal monitor divided by the number of metformin doses prescribed, and (2) scheduling or timing adherence, defined as the number of ingestible sensor tablets detected within a±2-hour time window around the pre-determined dosing time divided by the number of sensors detected. Descriptive statistics of daily glucose measurement, with mean, maximum, and minimum range, along with 95% confidence intervals across the study period were calculated.

### Verification of System and Data Cleaning

Patch function was checked using number of individual activity events reported over a 24-hour time period, with sampling every 5 minutes, that is, 288 measurements daily. The amount of continuous patch wearing per subject was recorded. In correlation calculations, days without full patch function were removed. The total number of events per subject per day recorded by the monitoring patch including heart rate, activity, and posture were approximately 720. Thus, over the 197 days of the study, data events recorded ranged from approximately 26,640-30,240 per subject, with a total number of data events recorded of greater than 141,840 for the entire study.

Algorithms were developed using Matlab and applied to analyze data for medication-taking behavior, sleep/rest, activity, heart rate, and then integrated with glucose measurements over (1) the entire study period, (2) daily, and (3) weekly. The sleep/rest state signal is derived from an algorithm based on activity, position, and pulse. The temporal structure of this sleep/rest state signal was used to derive the waking time. Waking time derivation was based on the following assumptions: (1) there is a single daily waking event, (2) short interruptions in long rest periods are transient state changes that should be ignored, and (3) similarly short rests in long waking periods do not suggest the main daily transitions. Visualizations were plotted using Matlab.

Pearson’s *r* correlation between sleep/rest metrics and medication-taking behavior was calculated using a single daily measure, derived from physiological features tracked by the personal monitor patch, and a daily adherence measure of either number of doses taken or timing of dose ingestion. A delay in medication ingestion of greater than 12 hours was considered to be a missed dose. The daily sleep/rest measure used were total hours of rest, and number of sleep disturbances between 10 p.m. and 8 a.m.

## Results

### Demographics and Traditional Summary Statistics of Medication-Taking and Timing Adherence

**Figure 2:**
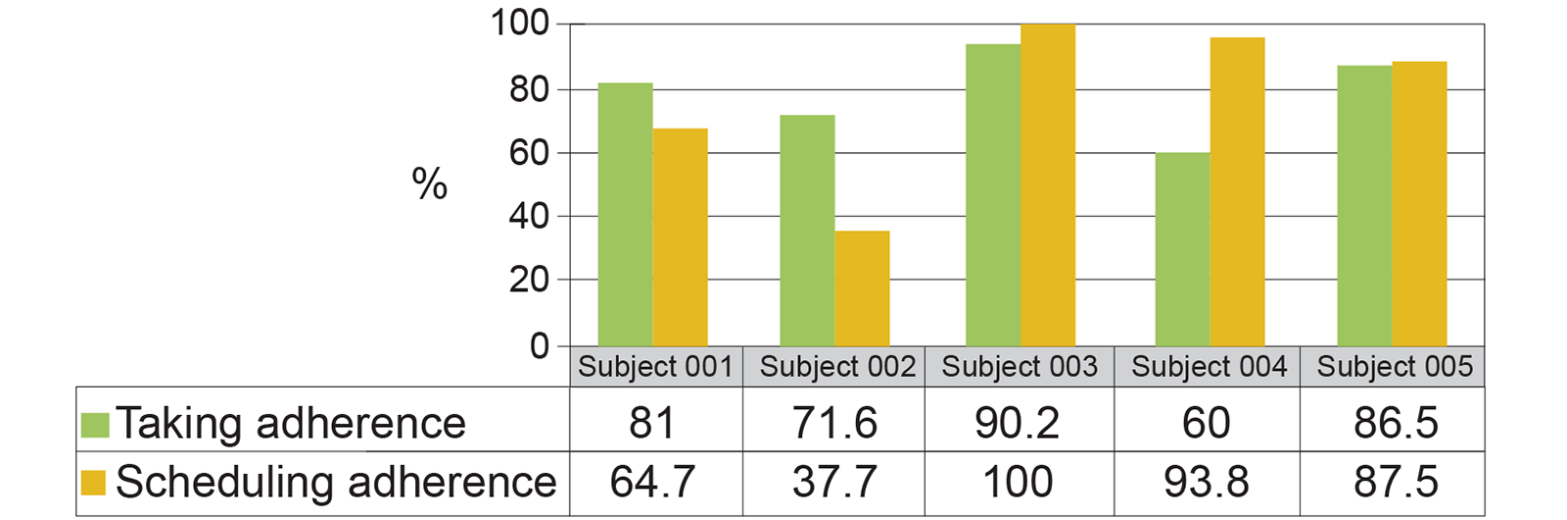
Standard representation of medication taking and scheduling adherence across study period by subject.

**Figure 3:**
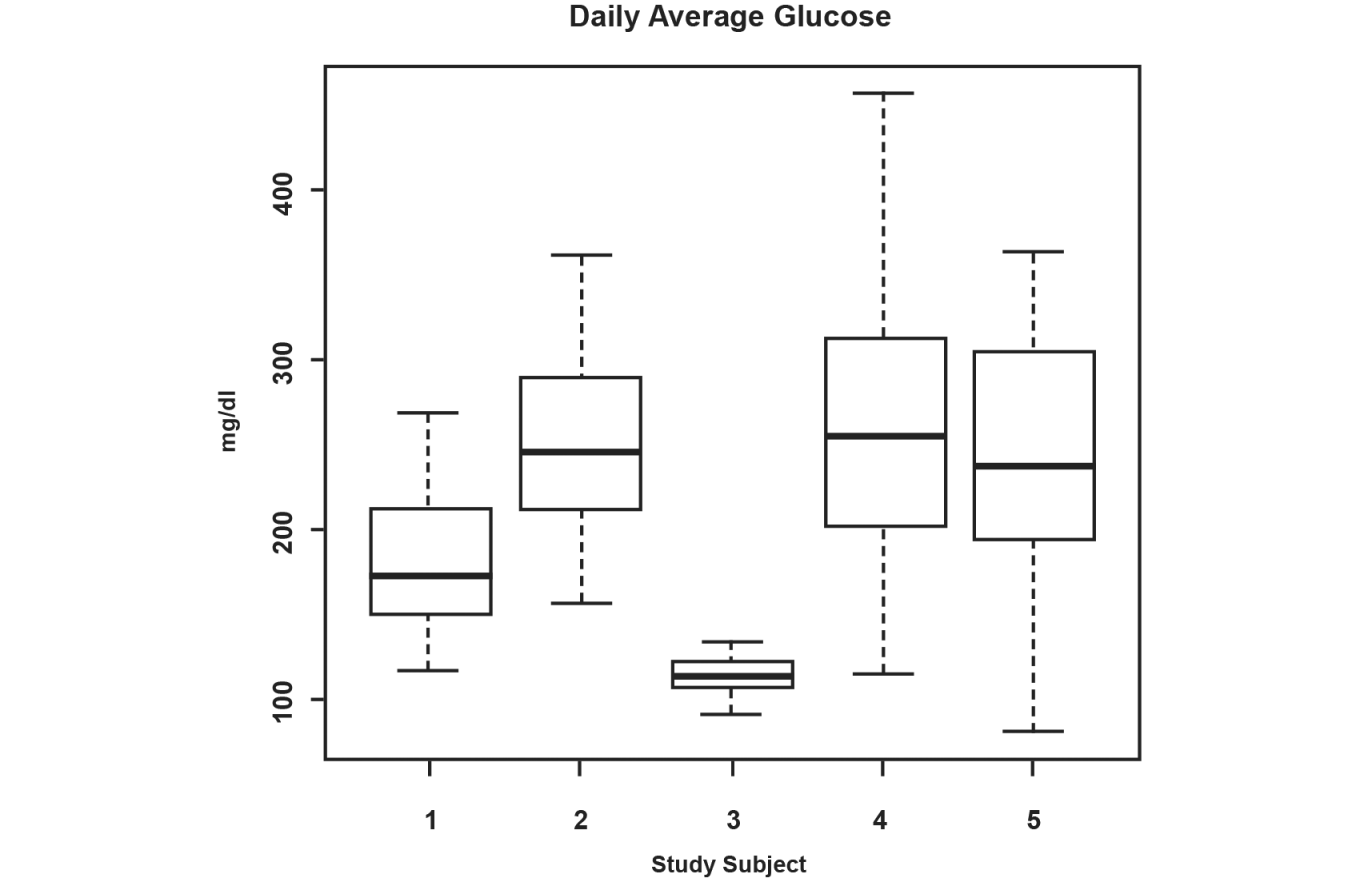
Daily average glucose measurements taken by subject.

Subjects were 5 patients with type II diabetes (2 females and 3 males), age 43-61, prescribed twice-daily metformin. Subjects 1, 2, and 5 used insulin in addition to metformin during the study. Days of participation per subject ranged from 37-42, with a total number of subject days of 197. On average, the cohort took 78% (SD 12) of prescribed medication and took 77% (SD 26) within the prescribed ±2-hour time window. Figure 2 shows those metrics for individual subjects. Figure 3 also shows the range of daily glucose measurements for each subject with the mean, maximum, minimum, and 95% confidence interval over the period of the trial.

### Verification of System and Data Cleaning

Patch use across 197 subject days of participation in the study showed subjects wore the patch continuously between 87-98% of the time. One isolated event of greater than 288 events was observed due to patch dysfunction. Data from days of no recording were removed as described above.

### Data Visualizations Incorporating the Entire Study Period

#### Medication-Taking Behavior, Timing of Ingestions, and Missed Doses

**Figure 4:**
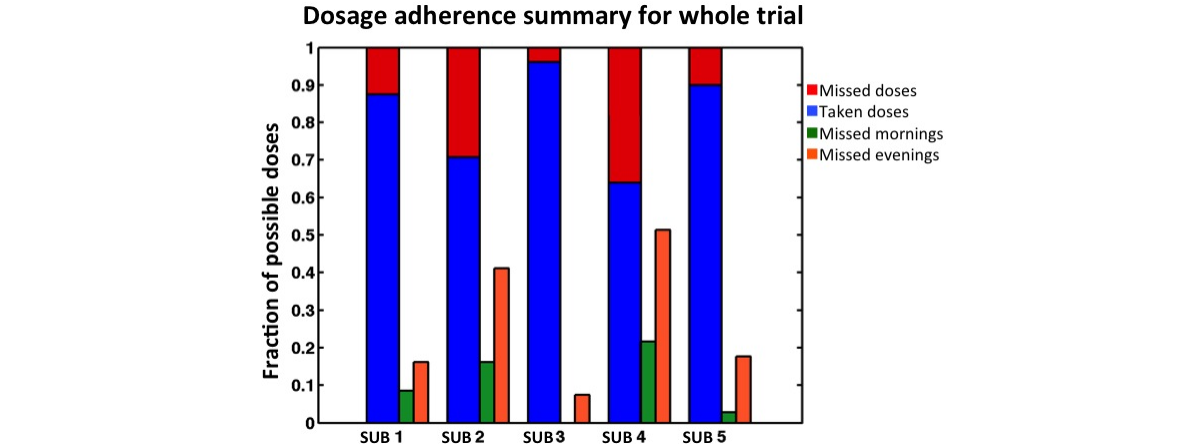
Dosage adherence summary for whole trial.

The DHFS system allows precise time-stamping of ingestions. The initial visualizations sort to provide a means to quickly evaluate how closely patients executed the twice daily dosing regimen prescribed and what the fraction of missed doses looked like in terms of the time of their occurrence. Figure 4 provides a summary graph of doses taken, total missed doses, and fraction of missed doses taking place in the morning or evening per subject over the period of the trial. The majority of missed doses occurred in the evening, particularly in Subjects 2 and 4 who had the lowest adherence.

**Figure 5:**
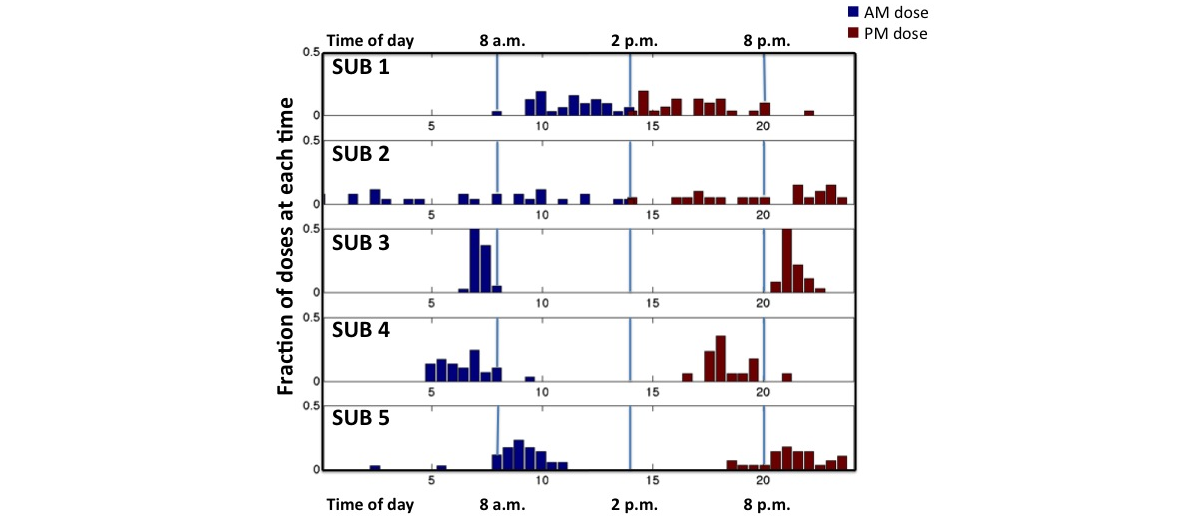
Frequency distribution of the time of day at which the subjects ingested medication.

In Figure 5, we plotted the fraction of medication ingested versus the time of day ingestion took place for the 5 subjects across the entire study period (Figure 5). This graphic provides information on the range of morning and evening medication ingestion timing and allows discernment of the subjects’ daily timing patterns. Subject 3 took medication over the narrowest range of time (in the morning over a 1.25 hour range and in the evening over a 2.4-hour range) and had a consistent pattern of ingestion. Subject 2 took medication over the widest range of times (over an 11-hour range in both the morning and evening) and had no consistently repeated pattern of ingestion timing in either the morning or the evening. Given that the mean half-life of metformin is approximately 5.7 hours in patients with type II diabetes [21], Subject 2 would have considerable variability in the plasma levels of metformin by timing adherence alone. Subject 4 took medication over a 4.5 and 4.8 hour time range, in the morning and evening respectively, but multiple doses were missed, mostly in the evening. Subjects 1 and 5 missed 19% and 14% respectively of their total doses. Subject 1 showed a range of ingestion times in the morning of 4.0 hours and a wide range of 11 hours for the timing of her second dose. Subject 5 ingested medication over a range of 8.5 hours in the morning and 5.8 hours in the evening. These graphs provide information on longitudinal patterns of missed doses and twice daily dose timing beyond the traditional summary graphs (Figure 2).

#### Waking Times, Medication-Taking Behavior, and Glucose Self-Monitoring

**Figure 6:**
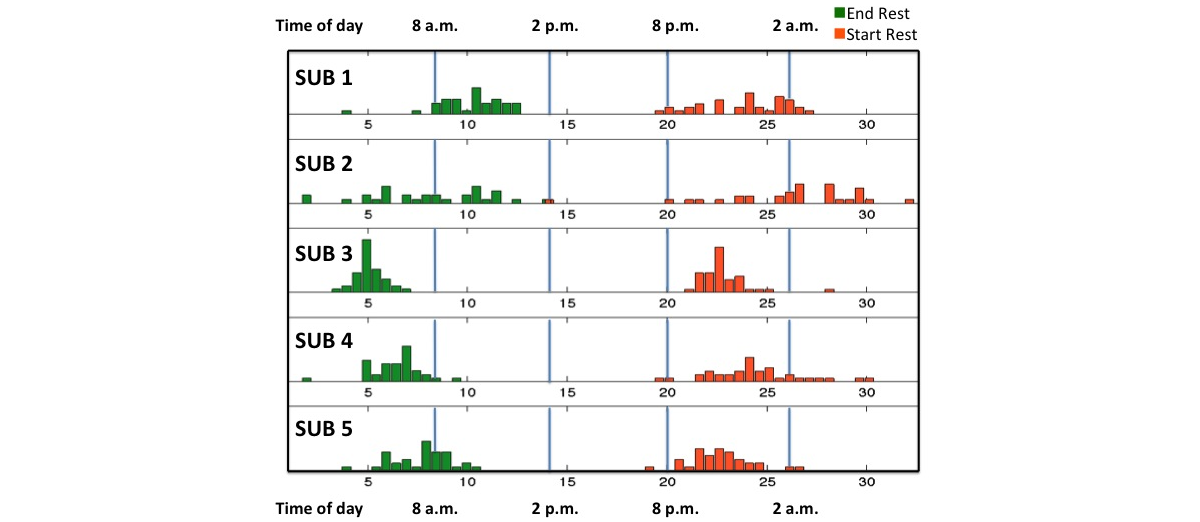
Frequency distribution of the estimated sleep and wake times of subjects.

The DHFS is capable of identifying sleep/rest behavior in addition to the precise timing of medication ingestion. The ability to execute consistent timing of drug ingestion twice daily may be connected to individual daily waking and bedtimes. Using DHFS data, we derived each subject’s waking time and bedtime (see Methods for mathematical derivation of these). Figure 6 shows the variability of sleep and waking time for each subject across the study.

We then visualized wake time with medication ingestion time for each subject, but also incorporated time of glucose measurement over the course of the study to explore if it was possible to visualize morning self-management patterns in individual subjects. Subjects in this study were instructed to take at least 1 daily glucose measurement, preferably first thing in the morning to provide a daily fasting glucose measure.

**Figure 7:**
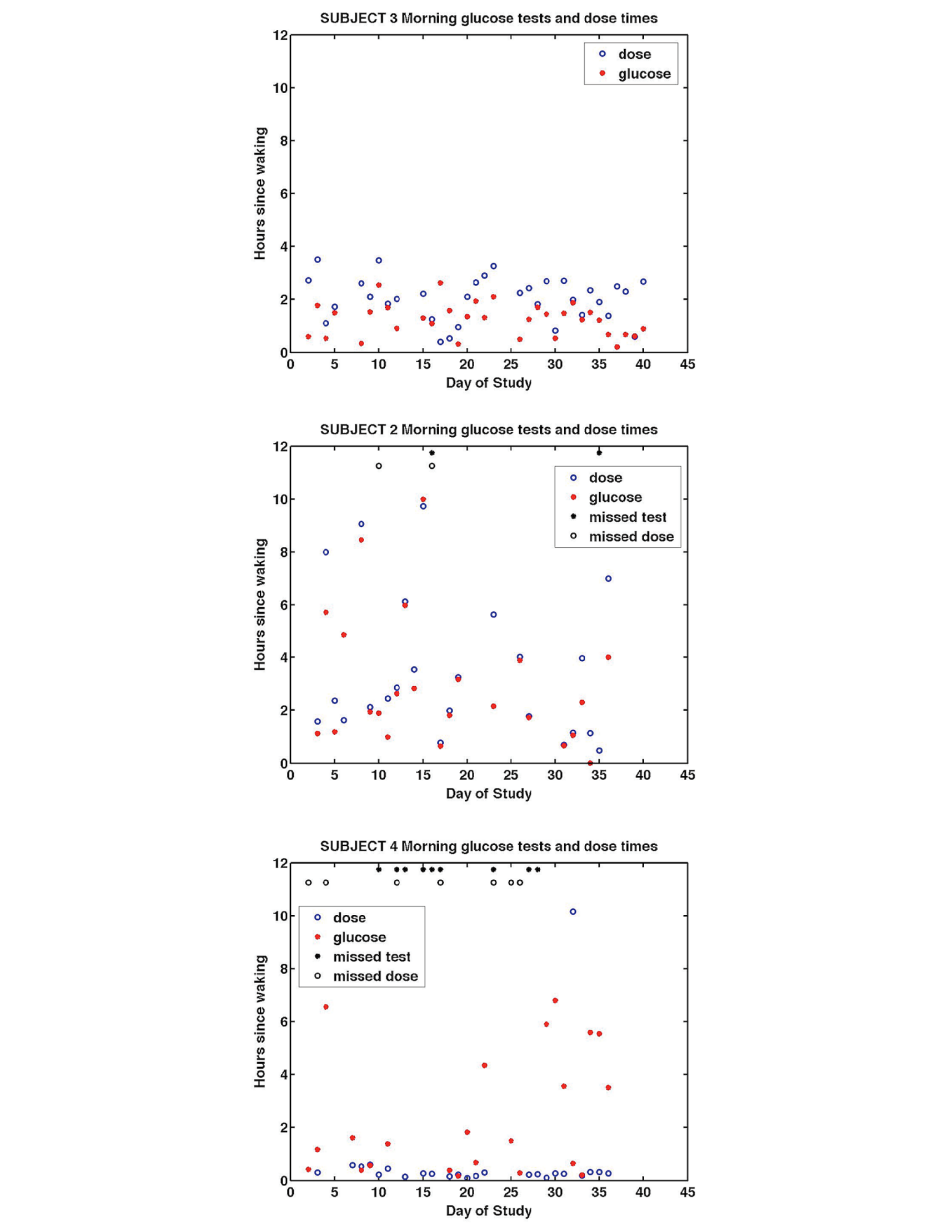
Morning glucose tests and metformin dose ingestion times in hours after waking for Subject 3 (top), Subject 2 (middle), and Subject 4 (bottom).

Figure 7 shows patterns of morning self-management of medication ingestion and blood glucose measurement in relation to waking time for Subjects 2, 3, and 4 (Subjects 1 and 5 are shown in Graphs 1 and 2 in Multimedia Appendix 1). Each individual subject displayed distinct patterns. Subject 3 ingested metformin within 3.5 hours of waking and always took blood glucose measurements before medication ingestion. Over the entire period of the trial, Subject 3 missed no morning doses. Subject 2 took medication and blood glucose measurements at widely varying times after waking but showed a very close association between timing of morning glucose measurement and medication ingestion (*r*=.6113, *P*>.001). Subject 4 took metformin within minutes of waking but missed multiple metformin doses. The proximity of waking time and medication ingestion for Subject 4 may partly account for the consistency in timing adherence of this subject, thus when the subject remembered to take the medicine, he did so on waking usually between 6-8 a.m. Subject 4 did not take blood sugar measures every morning and those taken were frequently made hours after waking and thus unlikely to represent fasting glucose values. Subjects 1 and 5 took their metformin dose within 4 hours of waking and their graphs are shown in Graphs 1 and 2 in Multimedia Appendix 1. The visualizations suggest that Subjects 2 and 4 did not have well-developed, consistent successful morning routines, in contrast to Subject 3. Subject 4 had some timing consistency that could be used to reinforce a medication ingestion routine on waking. Subject 2 showed no consistent pattern with waking. We note that these visualizations captured longitudinal patterns of waking, medication ingestion, and glucose measurement beyond traditional adherence summary graphs.

#### Activity Levels and Heart Rate

The DHFS also monitors heart rate and activity. An important aspect of patient self-management in chronic diseases like diabetes is maintaining healthy levels of physical activity. Subjects 3 and 4 had the most frequent and vigorous exercise levels, averaging between 8000 and 12,000 steps on most days. Subjects 1 and 2 had the lowest step counts averaging 4000-5000 steps per day. Subject 5 showed the widest range of daily step count with an average of 6000 steps per day. Step count distribution and daily average, maximum, and minimum heart rates for each patient are shown in Graphs 3 and 4 in Multimedia Appendix 1.

**Figure 8:**
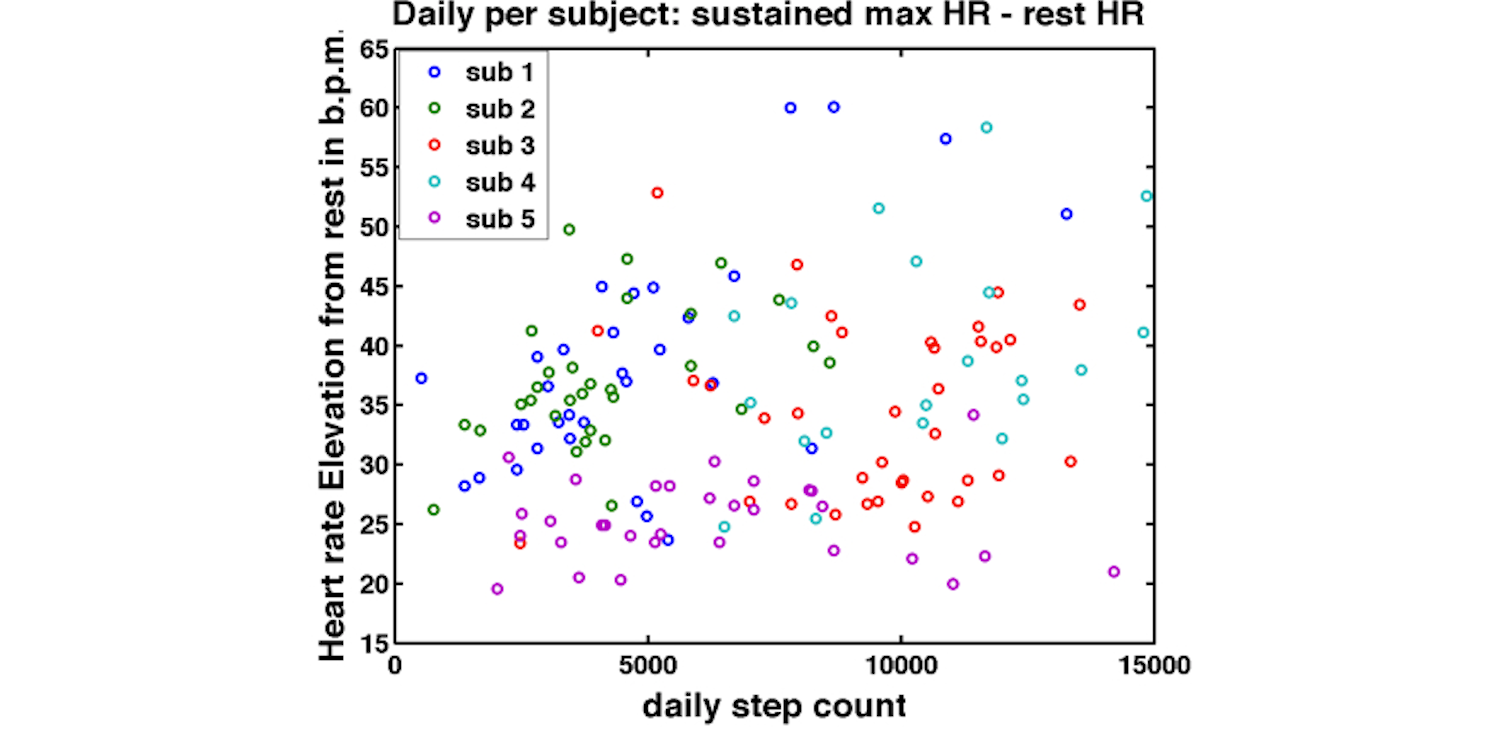
Sustained heart rate elevation from rest associated with daily step count, by subject. The graph plots the heart rate elevations from rest in beats per minute against each subject’s daily step counts.

We then combined data on heart rate and step activity to gain potential insight into the fitness levels of the subjects. We looked at the sustained maximum heart rate in terms of the elevation of the heart rate from resting (calculated for each individual) and its relationship to daily step count in each subject (Figure 8). In Figure 8, Subjects 1 and 2 have steep slopes in heart rate at relatively low step counts in comparison to Subjects 3 and 4. This may imply lower cardiovascular fitness levels in Subjects 1 and 2. Subject 5, a known hypertensive taking the medication atenolol, showed limited elevation in heart rate with elevated step count.

### Data Visualizations Over 24-Hour Periods

**Figure 9:**
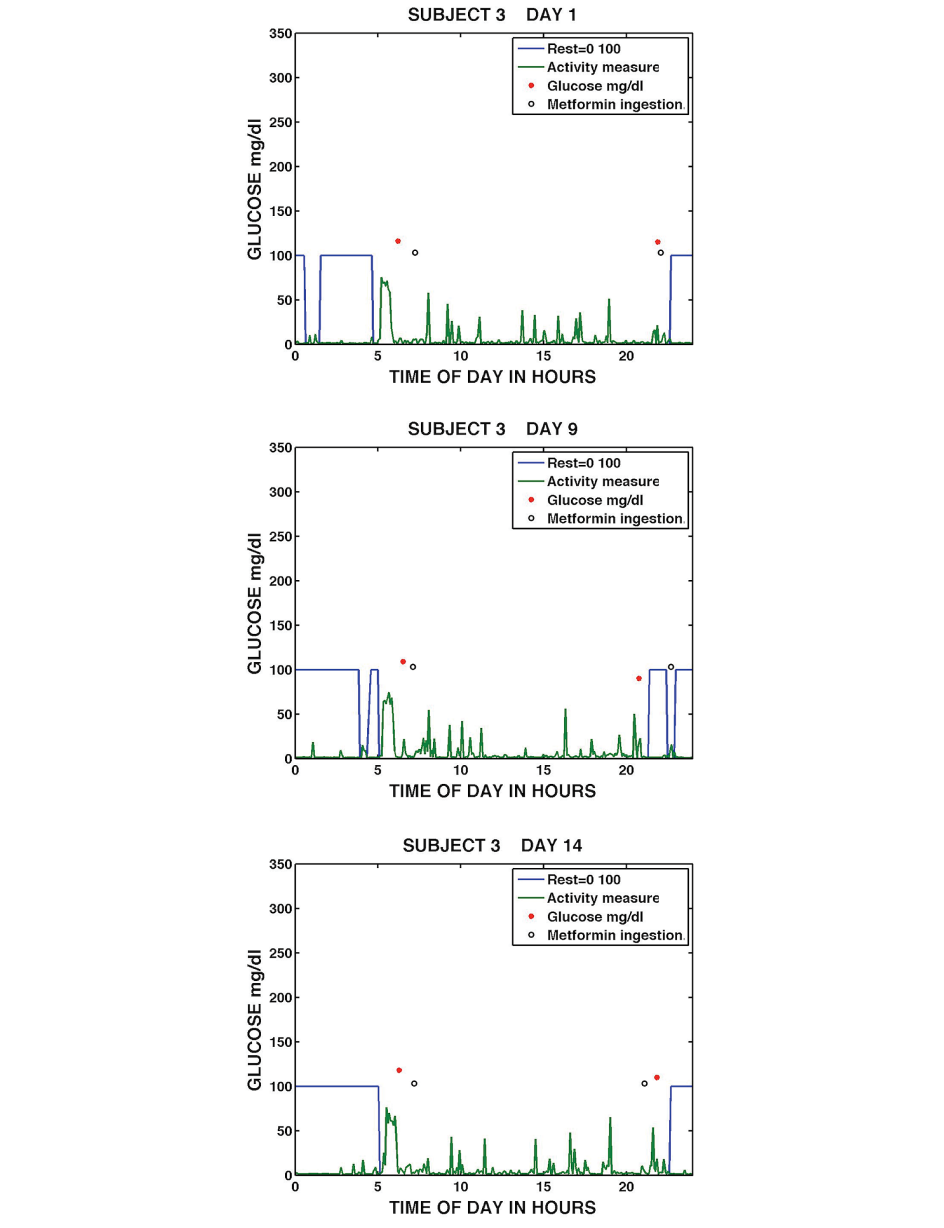
Daily medication taking, sleep/rest, activity, and glucose measurement for Subject 3 on Day 1 (top), Day 9 (middle), and Day 14 (bottom).

**Figure 10:**
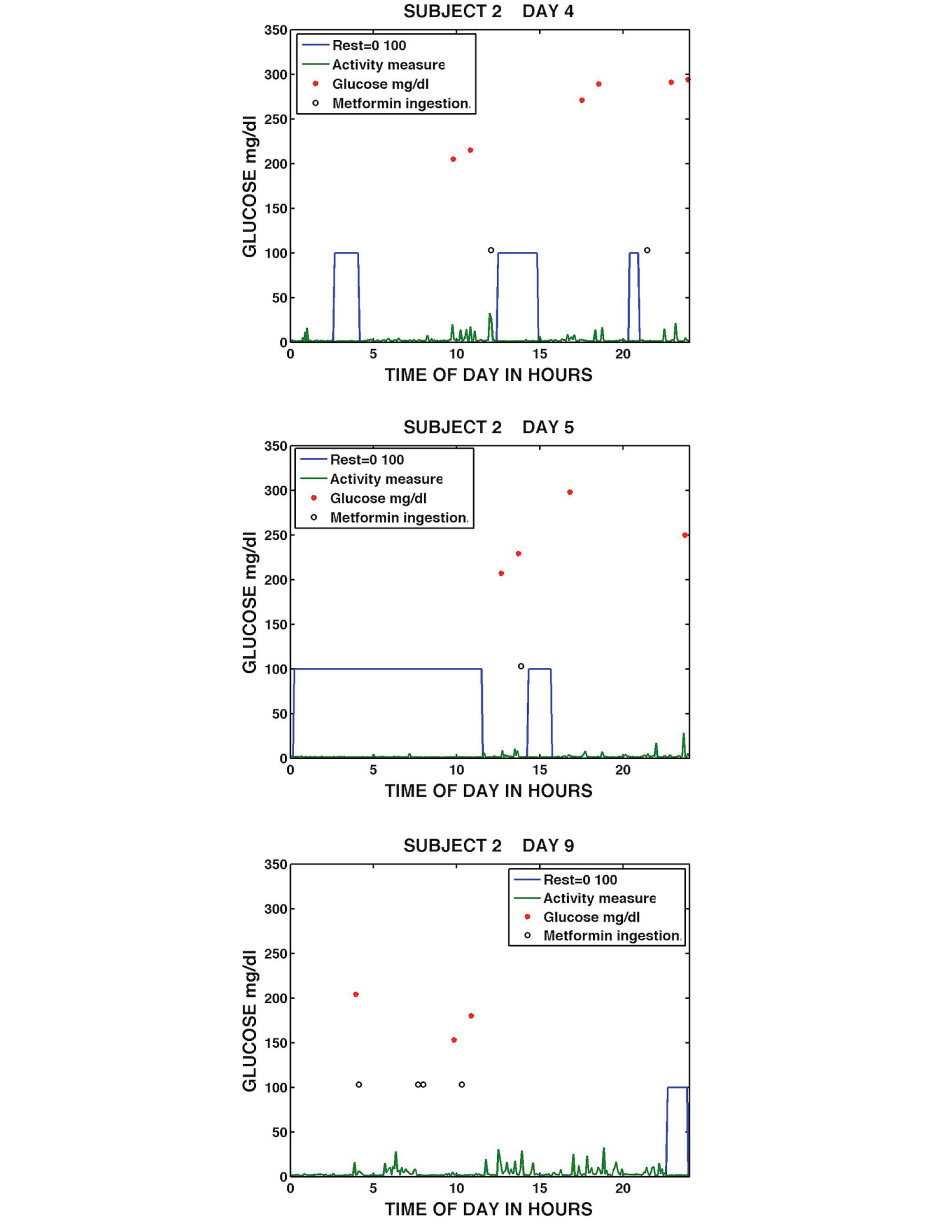
Daily medication taking, sleep/rest, activity, and glucose measurement for Subject 2, Day 4 (top), Day 5 (middle), and Day 9 (bottom).

**Figure 11:**
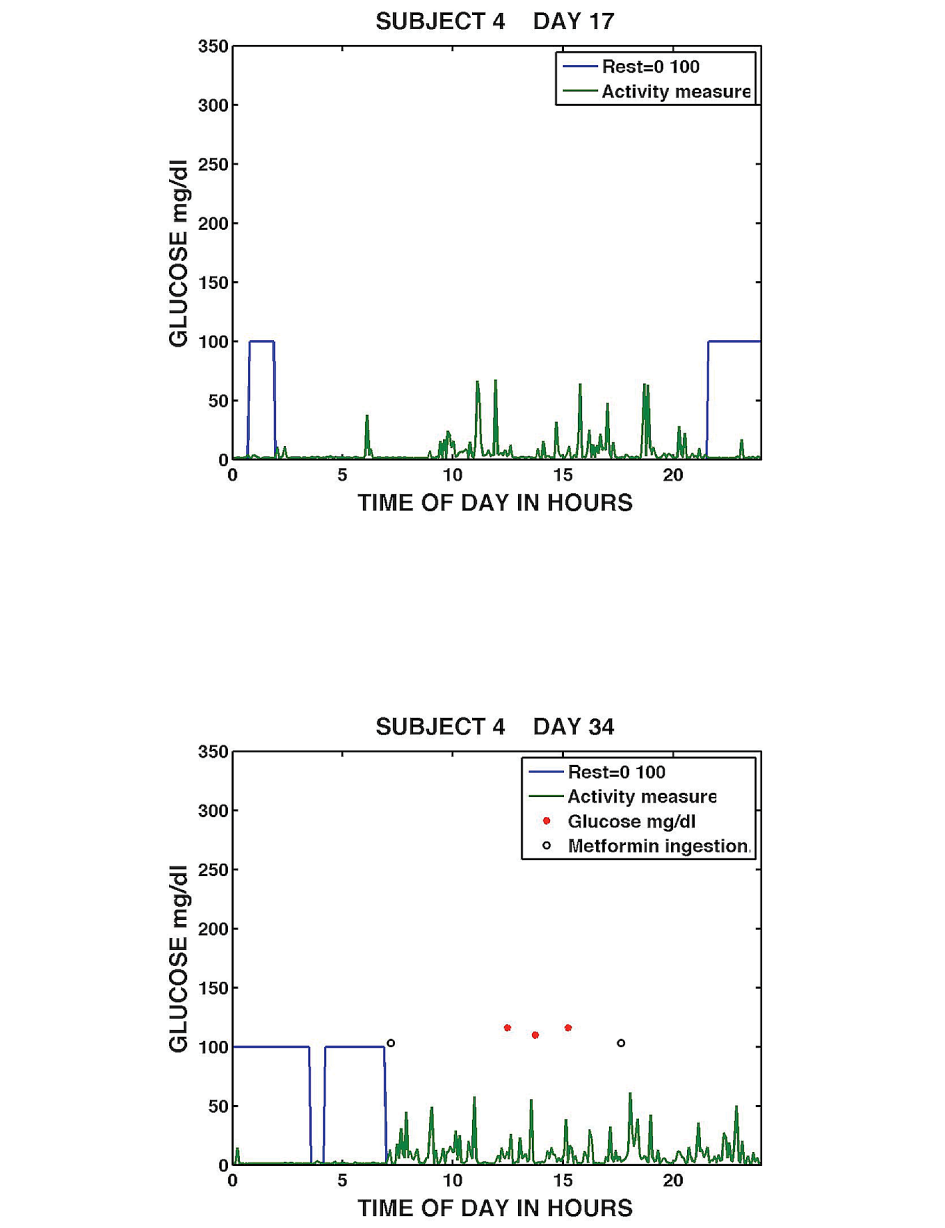
Daily medication taking, sleep/rest, activity, and glucose measurement for Subject 4, Day 17 (top), and Day 34 (bottom).

We then explored whether data analysis using visualization over a 24-hour time frame would offer different information than visualization over the entire study period. We generated graphs for each day of study participation, incorporating medication ingestion, sleep/rest, activity, and glucose measurement (both the time of day the glucose measurement took place and the actual glucose level are represented on the graph) for each subject. We found that with this combination of ingestion and physiological data, it was possible to generate visualizations that reconstructed the broad pattern of the patient’s day in relation to their medication-taking. Thus we could see when patients rose, tested their blood glucose, took their medication, became physically active, and later went to bed. Samples of these daily graphs for each subject are provided; Subjects 2, 3, and 4 are shown in Figures 9-11 and Subjects 1 and 5 in the Graphs 5 and 6 in Multimedia Appendix 1. These samples were chosen either because they represent a consistent feature of the subject’s longitudinal daily pattern or provided detail on how the structure of a patient’s day appeared related to their medication ingestion pattern that day.

Figure 9 shows visualizations of the most adherent participant, Subject 3 on Days 1, 9, and 14 of the study. The graphs show that Subject 3 had blocks of continuous sleep/rest (sleep/rest is visualized as a state that is either awake, 0, or asleep, 100), typically rose around 5 a.m., engaged in a period of the highest activity level, possibly exercise, subsequently took a blood glucose measurement, and ingested metformin medication around 7 a.m. The subject remained fairly active throughout the day, took a blood glucose measurement in the evening, promptly ingested metformin, and went to sleep/rest prior to midnight. Subject 3 showed limited variation in the patterns. One minor variation occurred in the evening of Day 9 when blood glucose was measured at approximately 9 p.m., followed by sleep/rest. Subject 3 then got up later to take metformin and then went back into sleep/rest at around 11 p.m. The visualizations capture the daily patterns of a highly adherent subject who demonstrates consistent self-management routines longitudinally over the study.

Figure 10 shows Subject 2 on Days 4, 5, and 9. Subject 2’s daily graphs showed considerable variation in behavior patterns. On Day 4, the subject’s continuous sleep/rest was of short duration taking place both at night and in the middle of the day; activity in between sleep/rest was low level; six blood glucose measurements were taken; and metformin was ingested at midday and then approximately 9 hours later. On the following Day 5, Subject 2 had an almost 12-hour period of sleep/rest starting just after midnight, took her blood glucose around noon, took metformin dose around 2 p.m., and had a further period of sleep/rest. The subject had low activity levels outside sleep/rest periods and missed the evening metformin dose. On Day 9, Subject 2 had no identifiable sleep/rest period until late evening, again showed low levels of activity, measured her blood glucose twice, and took four metformin in a 24-hour period. Two of these metformin were taken minutes apart. Subject 2’s graphs showed wide daily variation, with continuous changes in sleep/rest patterns and erratic medication-taking behavior. These visualizations provide considerable data beyond traditional adherence summary statistics. It can be appreciated that poor adherence in this subject may not respond to simple medication reminders or even missed dose reminders but will require a broader approach. The visualizations suggest health care providers should talk to the subject more broadly about factors that influence medication adherence such as sleep disturbance, mood alteration associated with significant sleep disturbance, the length of time these issues have persisted and should extend the interview to try to understand whether the patient’s daily patterns were the result of untreated concurrent conditions such as depression, hyperthyroidism, chronic pain, gastroesophageal reflux disease, or were a reaction to external stressors or both.

Figure 11 shows sample daily graphs from Subject 4 on Days 17 and 34. Subject 4 appeared to show some variability in medication-taking with variable sleep length. On Day 17, Subject 4 had poor sleep/rest and did not take a metformin or a blood glucose measurement. On Day 21, after a continuous period of sleep/rest, Subject 4 took a morning dose of metformin prior to measuring his blood glucose three times between noon and 3 p.m. and took an evening dose between 6 and 7 p.m. The graphs provide examples of a day when the patient is non-adherent and a day when the patient was adherent. (Samples and discussion of daily graphs for Subjects 1 and 5 are available as Graphs 5 and 6 in Multimedia Appendix 1).

This interval of analysis enabled highly individualized insight into each participant’s daily behavior patterns over the period of study in relationship to medication ingestion. The data potentially provide information to support individually tailored interventions to improve adherence outcomes.

### Data Visualizations Over Weekly Periods

**Figure 12:**
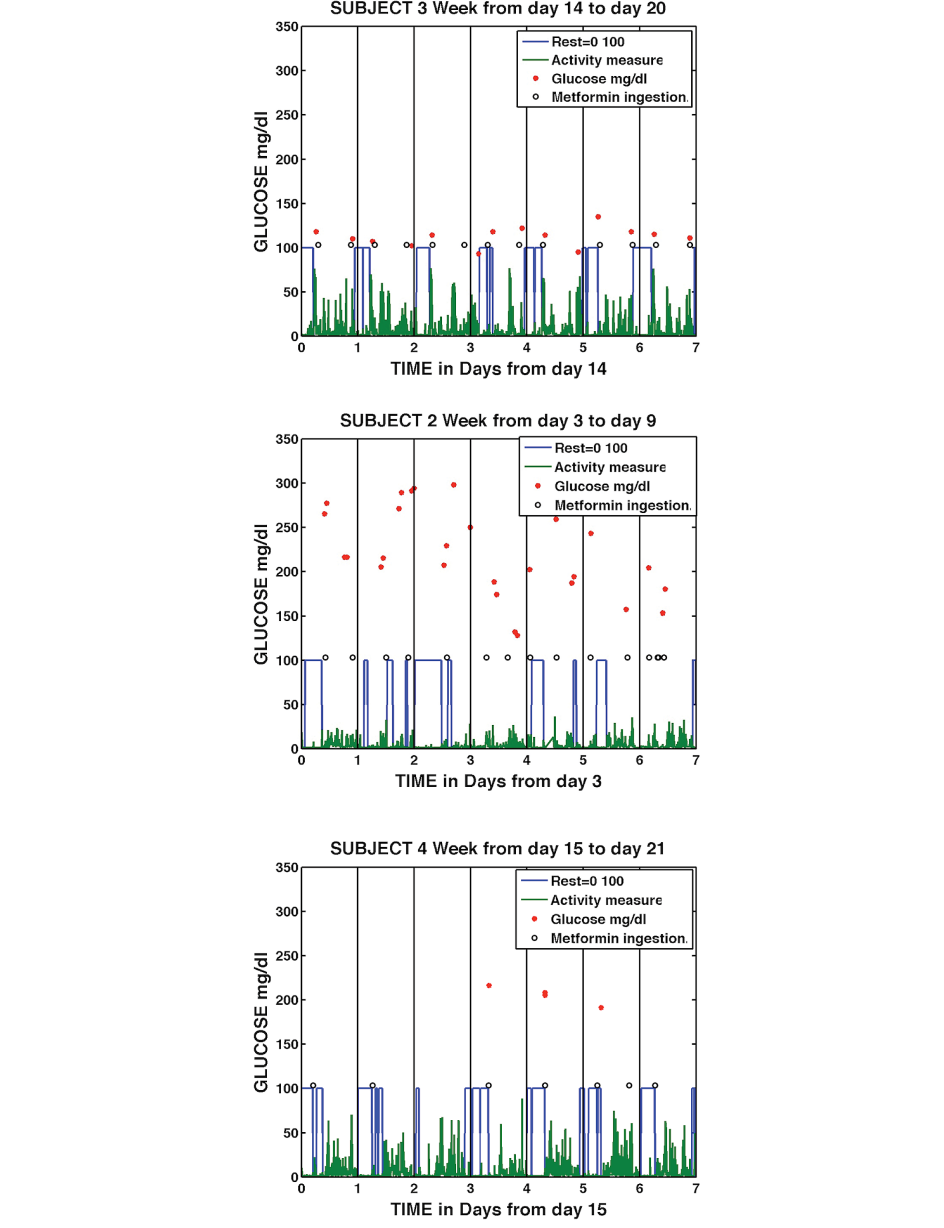
Weekly medication taking, sleep/rest, activity, and glucose measurement for Subject 3, Days 14-20 (top); Subject 2, Days 3-9 (middle); and Subject 4, Days 15-21 (bottom).

While daily graphs demonstrated the ability of the DHFS to provide detailed insights into individual daily patterns of behavior, we wanted to capture both the detail of the daily analysis with longitudinal patterns of behavior, so we generated visualizations using the data input over weekly periods. Figure 12 show samples of graphs combining medication ingestion, sleep/rest, activity, and glucose measurement (both the time of day the glucose measurement took place and the actual glucose level are represented) over weekly time frames. The sample graphs chosen either represent the typical longitudinal pattern of that subject over the course of the study or include patterns associated with incomplete medication-taking. Figure 12 shows examples of these, for Subjects 2, 3, and 4. (Weekly graph examples for Subjects 1 and 5 are available as Graphs 7 and 8 in Multimedia Appendix 1). Figure 12 shows Subject 3 over a 1-week period from Days 14-20, with regular periods of sleep/rest, exercise, active daytime periods, and glucose measurements taken twice daily with all readings below 150 mg/dl. Subject 2 is shown over a 1-week period from Days 3-9, with erratic and often interrupted sleep/rest patterns, four medication dose ingestions on Day 9, varying numbers of blood glucose measurements, mostly greater than 180 mg/dl, and generally low levels of activity. Subject 4 is shown on Days 15-21 of the trial, with regular activity, variable sleep/rest length, and missed multiple medication doses, particularly in the evening. Subject 4 took four blood glucose measurements during this period and had elevated glucose readings.

#### Analysis of Sleep/Rest and Medication-Taking Behavior

The daily and weekly visualizations suggested there may be some association with variation in sleep pattern and medication ingestion in some subjects, particularly Subject 2. To evaluate whether there was a relationship between sleep/rest and medication-taking behavior in the individual patients, we looked at the correlation between (1) total hours of sleep/rest the previous night and pill taking the next day, and (2) sleep/rest disruptions (breaks in sleep between 10 p.m. and 8 a.m.) and medication-taking behavior the following day for each subject over the entire study period. Tables 1 and 2 show these correlations. The medication-taking behavior of Subjects 2 and 4 appeared to be significantly affected by the amount of sleep/rest they had the previous night. Subject 2 also appeared to have a robust relationship between the absence of breaks in sleep/rest between 10 p.m. and 8 a.m. and the likelihood of taking metformin medication the following day. Larger studies are needed to fully evaluate the relationship between amount of sleep/rest and medication adherence.

**Table 1:** Total hours of sleep/rest the previous night correlated with medication-taking adherence the next day.

Subject	Correlation coefficient (*r*)	*P* value
1	-.158	.433
2	.475	.022^a^
3	.206	.236
4	.350	.050^a^
5	.188	.321

^a^ *P*<.05

**Table 2:** Number of breaks in sleep/rest between 10 p.m. and 8 a.m. correlated with medication-taking adherence the next day.

Subject	Correlation coefficient (*r*)	*P* value
1	-.143	.427
2	-.468	.009^a^
3	.136	.424
4	-.116	.506
5	.066	.722

^a^ *P*<.05

## Discussion

### Principal Findings

The DHFS is the only system to date that can detect actual medication ingestion events. It also captures dense physiological data simultaneously. This study aimed to use modern methods of visual analytics to represent continuous and discrete data from this novel sensor system, plotting multiple different data types simultaneously to evaluate the potential of the DHFS to capture longitudinal patterns of medication adherence. The DHFS was able to provide remote monitoring of ingestion events and physiological metrics in the natural setting over the entire period of the study. High levels of continuous patch wearing with an early prototype were demonstrated in this study (current monitor patch versions are four generations from the version used).

Our study demonstrated a number of findings. Visualization of the data with simultaneous variable plotting, integrating physiologic and medication ingestion data, provided useful detailed information on longitudinal medication-taking and timing adherence patterns beyond traditional summary statistics. Varying the time interval over which the variables were visualized provides complementary data, with visualizations over a 24-hour period demonstrating the ability to reconstruct basic daily behavior patterns in relation to medication ingestion. While there was some overlap of behavior in this small sample, essentially each subject emerged as having their own distinct longitudinal pattern. Even if that pattern was one of variability, such as Subject 2, who showed widely variable patterns of medication ingestion and timing with highly variable morning and daily routines, including sleep/rest patterns. In contrast, our analyses also enabled us to look at the behavior patterns of Subject 3, a patient with type II diabetes, who was highly adherent to the prescribed regimen and whose glucose measures remained under 150 mg/dl over the period of the study. The regularity of this subject’s daily routine, including sleep/rest cycles and level of exercise, was striking in comparison to some of the other subjects. As the number of these types of analyses using the DHFS over wide populations grows, it will be also be important to increase our understanding of how highly adherent and optimally self-managed patients behave. This will enable us to identify “success habits” and share those with other patients. The focus of adherence consultations can then include not just “how you are failing,” but also generalized examples of “how to succeed.”

One of the most significant strengths of the visualizations presented is that they encompass idiographic data analysis techniques that can focus on individual variability over time. These methods are uniquely suited to the analysis of individually tailored interventions evolving over time to promote medication adherence. The initial graphs on timing of ingestions and missed doses may allow the health care provider and patient to discuss patterns of medication-taking across a monitored time period and to focus on the time of day patients may need the most support. Addition of the 24-hour graphs provide detailed information on patterns of activity and sleep/rest on days when doses were missed and may allow a more in-depth interaction with the patient on the types of support needed. To illustrate how this information could be used in practice, in discussion with Subject 2, the health care provider could extend the interview on factors affecting this patient’s medication adherence to include sleep/rest patterns and other disorders associated with significant sleep disturbance, such as depression, hyperthyroidism, chronic pain, gastroesophageal reflux disease, to try to understand whether the patients’ daily patterns are the result of untreated concurrent conditions or a reaction to stressors, or both. The visualizations provide the opportunity to extend the health care patient interview allowing broader personalized intervention to support treatment success beyond the use of medication reminders alone, which would be unlikely to successfully support medication adherence in Subject 2. However, Subject 4, who most frequently took his medication after waking, may well respond to reminders alone, particularly missed dose reminders, timed around his waking. Similarly dose reminders may work well in the evening time for Subjects 1, 4, and 5, particularly missed medication reminders around dinner time, to reduce the number of missed medications in the evening. The fully developed DHFS is capable of delivering near real-time feedback, which can be incorporated into individually tailored patient missed-dose reminder interventions. Thus the visualizations tell individual stories for each subject that can inform intervention and future management to support medication adherence and patient self-management.

A major stated aim of this paper is to provide and explore modes of data visualization that are accessible to both health care worker and patient consumer. This requires visualizations to preserve the accuracy of the data, while maintaining accessibility to the maximum number of individuals without prior training. Despite the density of the multiple different data types summarized by some of the visualizations, it was possible to develop graphs that could be easily understood and potentially suitable for use by patients and health care workers with minimal training. Health care workers in particular have very little time, and continuous data streaming of individual variables can easily overwhelm providers with data that may be largely unusable. As stated in the introduction, visual analytics is an emerging discipline that has shown significant promise in addressing many of these information overload challenges [18]. In data-rich illustrations, every data point has value, when such illustrations are examined closely [19]. Further, as Tufte emphasizes, a key take-away point is that data-rich graphics do not simply represent the data in visual form but can reveal what the data mean [19]. We contend that an example of this principle is the multiple data stream visualizations over 24 hours, where only by visualizing this data could we appreciate that we were able to reconstruct the broad structure of a person’s day and see how their medication-taking behavior was related. Plotting data streams together, providing a data-rich representation, was more meaningful than plotting each data stream individually and revealed information that would not have been appreciated if each data stream had been plotted individually.

Based on visualizations showing variability in sleep/rest and medication-taking behavior in some subjects, we evaluated potential correlations between medication-taking behavior sleep/rest patterns. This analysis suggested medication-taking behavior in 2 individual subjects was significantly influenced by sleep/ rest patterns over the period monitored. Other researchers have found sleep disturbance, especially when reported with depression, is associated with poor medication adherence [22,23]. Larger studies over longer periods of time would be needed to definitively establish whether sleep disturbance is significantly correlated with non-adherence in certain individuals and in general.

As these visualizations could prove useful and relevant in clinical practice, investigation of their ease of use and utility to health care workers and patients is currently in progress. While our analyses across varying time intervals provided complementary data, using a weekly interval of analysis we were able to provide much of the detail of daily structure in combination with longitudinal patterns over a 1-week period. Thus, we estimate that the weekly visualizations will prove the most useful interval of evaluation in future studies incorporating tailored individual interventions to support medication adherence and optimal self-management. The addition of subject input such as personal event logs would further increase the impact of these visual analyses of DHFS data and enrich feedback that could be given to individuals to guide self-management.

In future, as the number of these analyses and use of the DHFS system grows, reference databases on patterns of longitudinal medication adherence and self-management patterns will accumulate and measurable estimates of comparability of longitudinal patterns to large sample populations will be available. Ultimately, we anticipate that machine-learning techniques will make predictive interventions possible to support longitudinal medication adherence and patient self-management.

### Limitations

The major limitation of this work is the study size. Work is currently underway to further refine analytic algorithms within larger studies. Co-ingestion of an edible sensor whenever taking metformin, as occurred in this user experience study, is now being replaced with combined formulations of edible sensor and medication [24]. The focus of this work is analysis of medication-taking and physiological correlates. Limited comment is made here on the adequacy of glycemic control of type II diabetes in these subjects. Further, rigorously designed and larger studies will be necessary to evaluate the DHFS as a tool for monitoring and improving glycemic control within type II diabetic self-management.

### Conclusions

Visualizations integrating medication ingestion and physiological data from the DHFS over varying time intervals captured detailed individual longitudinal patterns of medication adherence and self-management in the natural setting. Visualizing multiple data streams simultaneously, providing a data-rich representation, revealed information that would not have been appreciated by plotting data streams individually. Such analyses provided data far beyond traditional adherence summary statistics and may form the foundation of future personalized predictive interventions to drive longitudinal adherence and support optimal self-management in chronic diseases such as diabetes.

## Appendix

Multimedia Appendix 1 Supplementary graphs.

## Abbreviations

DHFS: Digital Health Feedback System
FDA: Food and Drug Administration

## References

[1] Osterberg L, Blaschke T (2005). Adherence to medication. N Engl J Med.

[2] Standing Committee of European Doctors (2011). Background Briefing.

[3] Ernst FR, Grizzle AJ (2001). Drug-related morbidity and mortality: updating the cost-of-illness model. J Am Pharm Assoc (Wash).

[4] New England Healthcare Institute (2009). Research Brief. New England Healthcare Institute - August.

[5] Aronson JK (2007). Compliance, concordance, adherence. Br J Clin Pharmacol.

[6] Laine C, Newschaffer CJ, Zhang D, Cosler L, Hauck WW, Turner BJ (2000). Adherence to antiretroviral therapy by pregnant women infected with human immunodeficiency virus: a pharmacy claims-based analysis. Obstet Gynecol.

[7] Bova CA, Fennie KP, Knafl GJ, Dieckhaus KD, Watrous E, Williams AB (2005). Use of electronic monitoring devices to measure antiretroviral adherence: practical considerations. AIDS Behav.

[8] Wendel CS, Mohler MJ, Kroesen K, Ampel NM, Gifford AL, Coons SJ (2001). Barriers to use of electronic adherence monitoring in an HIV clinic. Ann Pharmacother.

[9] Olivieri NF, Matsui D, Hermann C, Koren G (1991). Compliance assessed by the Medication Event Monitoring System. Arch Dis Child.

[10] Denhaerynck K, Schäfer-Keller P, Young J, Steiger J, Bock A, De GS (2008). Examining assumptions regarding valid electronic monitoring of medication therapy: development of a validation framework and its application on a European sample of kidney transplant patients. BMC Med Res Methodol.

[11] Hafezi H, Robertson T, Moon G, Au-Yeung K, Zdeblick M, Savage G (2015). An ingestible sensor for measuring medication adherence. IEEE Trans Biomed Eng.

[12] Eisenberger U, Wüthrich RP, Bock A, Ambühl P, Steiger J, Intondi A, Kuranoff S, Maier T, Green D, DiCarlo L, Feutren G, De GS (2013). Medication adherence assessment: high accuracy of the new Ingestible Sensor System in kidney transplants. Transplantation.

[13] Au-Yeung KY, Moon GD, Robertson TL, Dicarlo LA, Epstein MS, Weis SE, Reves RR, Engel G (2011). Early clinical experience with networked system for promoting patient self-management. Am J Manag Care.

[14] DiCarlo L, Moon G, Intondi A, Duck R, Frank J, Hafazi H, Behzadi Y, Robertson T, Costello B, Savage G, Zdeblick M (2012). A digital health solution for using and managing medications: wirelessly observed therapy. IEEE Pulse.

[15] Kane JM, Perlis RH, DiCarlo LA, Au-Yeung K, Duong J, Petrides G (2013). First experience with a wireless system incorporating physiologic assessments and direct confirmation of digital tablet ingestions in ambulatory patients with schizophrenia or bipolar disorder. J Clin Psychiatry.

[16] Thomas JJ, Cook KA, National V, Analytics C (2005). Illuminating the path.

[17] West VL, Borland D, Hammond WE (2015). Innovative information visualization of electronic health record data: a systematic review. J Am Med Inform Assoc.

[18] Caban JJ, Gotz D (2015). Visual analytics in healthcare--opportunities and research challenges. J Am Med Inform Assoc.

[19] Tufte E (2001). The visual display of quantitative information.

[20] Au-Yeung K, DiCarlo L, Ionescu A, Duong J, Moon G, Littlewort G (2012). First user experience of a comprehensive monitoring system to facilitate self-management of diabetes.

[21] Hong Y, Rohatagi S, Habtemariam B, Walker JR, Schwartz SL, Mager DE (2008). Population exposure-response modeling of metformin in patients with type 2 diabetes mellitus. J Clin Pharmacol.

[22] Gay C, Portillo CJ, Kelly R, Coggins T, Davis H, Aouizerat BE, Pullinger CR, Lee KA (2011). Self-reported medication adherence and symptom experience in adults with HIV. J Assoc Nurses AIDS Care.

[23] Phillips KD, Moneyham L, Murdaugh C, Boyd MR, Tavakoli A, Jackson K, Vyavaharkar M (2005). Sleep disturbance and depression as barriers to adherence. Clin Nurs Res.

[24] Browne S, Haubrich R, Campbell R, DiCarlo L, Peloquin C, Moser K (2014). Wirelessly observed therapy (WOT) compared to directly observed therapy (DOT) for the treatment of tuberculosis.

