# Supplementary material for: Let Visuals Tell the Story: Medication Adherence in Patients with Type II Diabetes Captured by a Novel Ingestion Sensor Platform
Source: JMIR Mhealth Uhealth. 2015 Dec 31;3(4):e108. doi: 10.2196/mhealth.4292 (PMC4713908; doi:10.2196/mhealth.4292)
Supplement: Multimedia Appendix 1 [file mhealth_v3i4e108_app1.pdf]

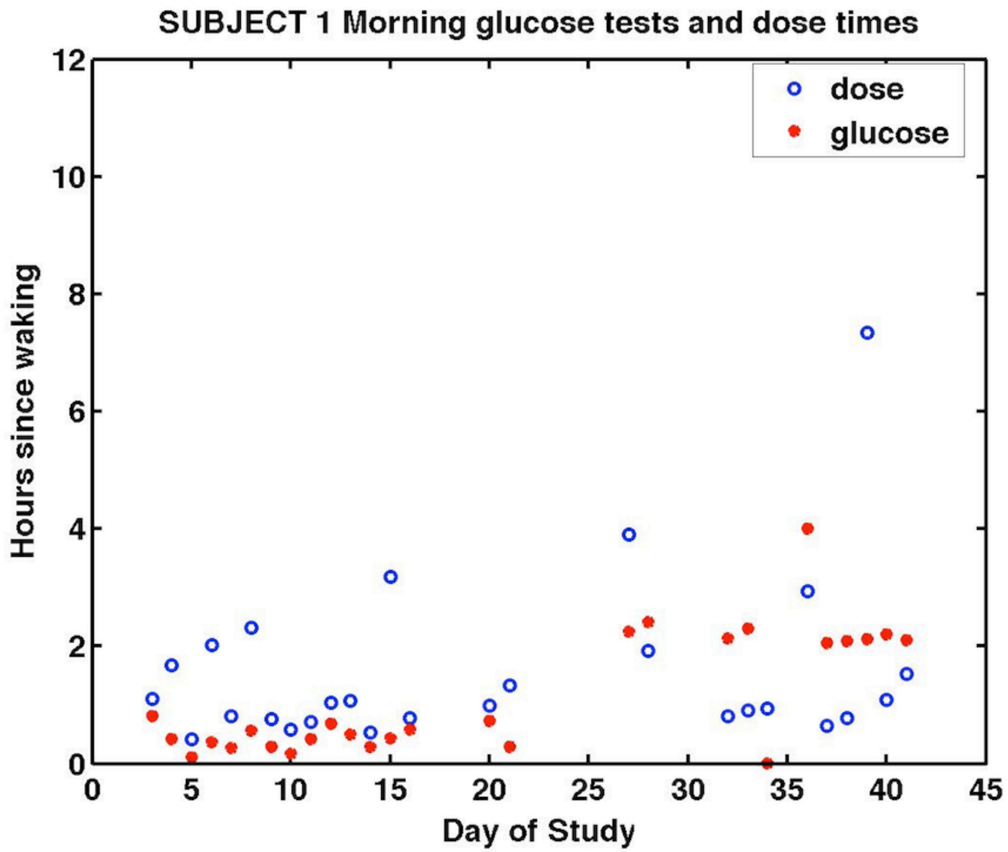

Graph 1: Morning glucose tests and metformin dose times for Subject 1.

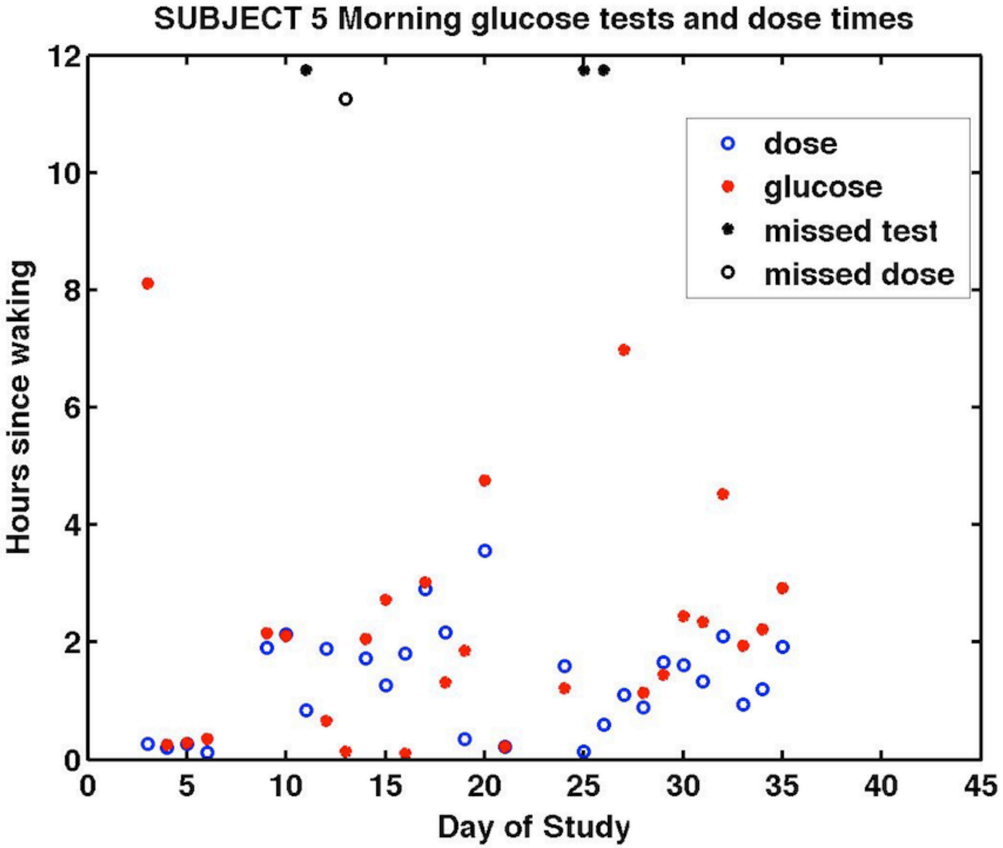

Graph 2: Morning glucose tests and metformin dose times for Subject 5.

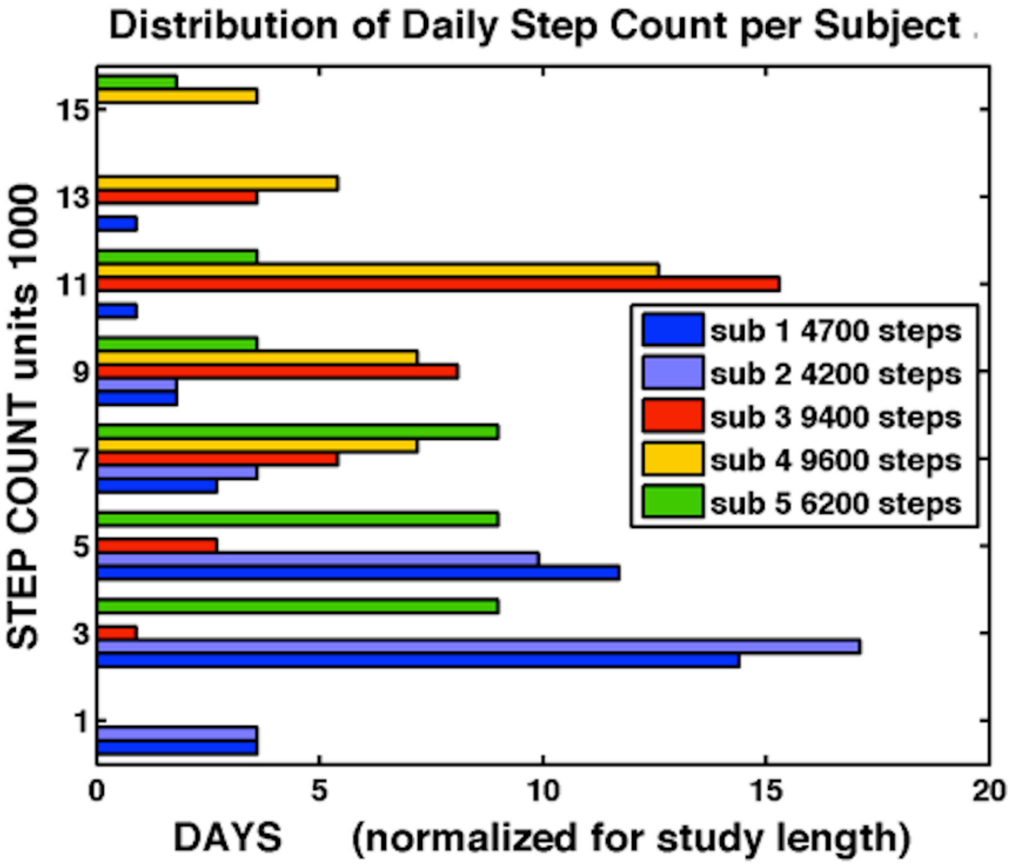

Graph 3: Step count distribution for each subject.

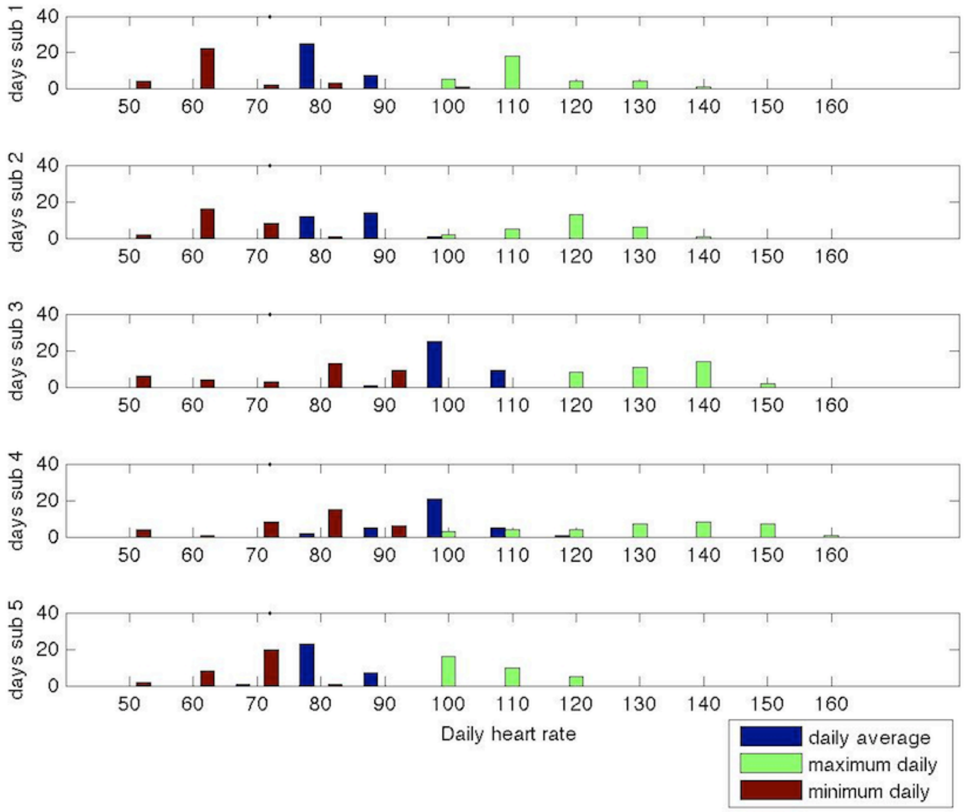

Graph 4: Heart rate variability by subject.

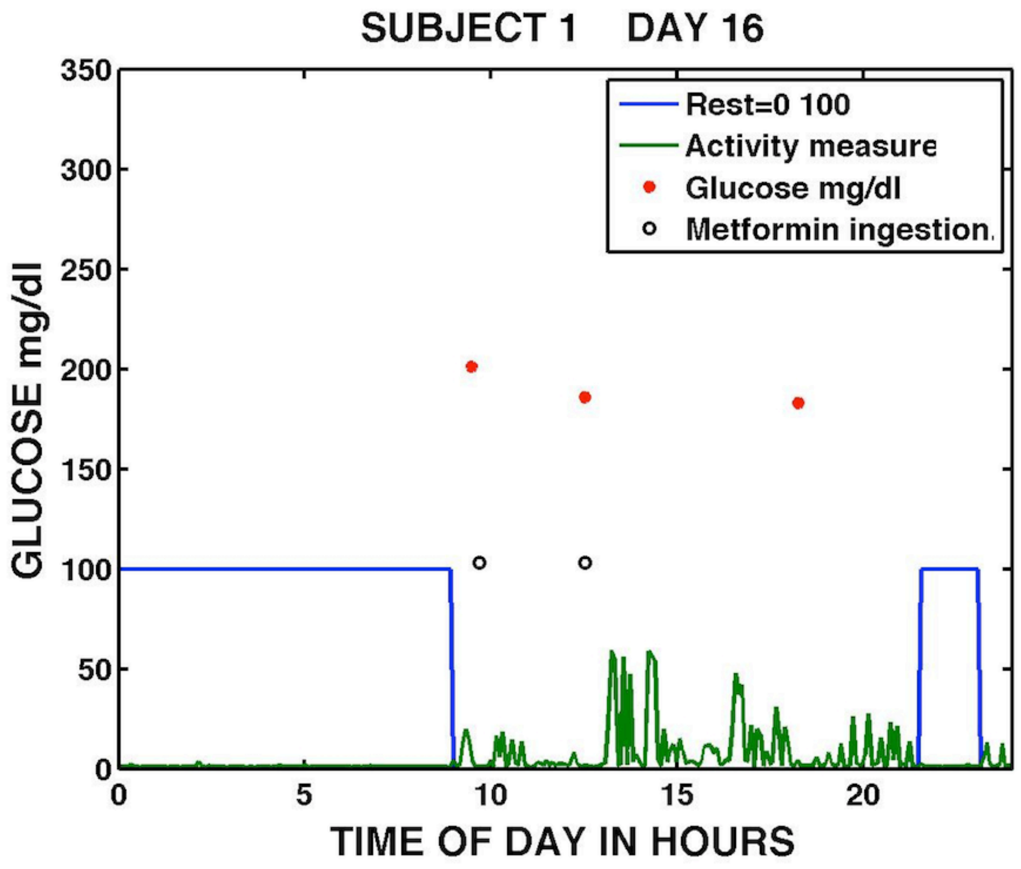

Graph 5: Daily medication taking, sleep/rest, activity, and glucose measurement for Subject 1, Day 16. Graph shows continuous block of sleep until approximately 9.00am with a glucose measurement and metformin ingestion prior to 10.00am. A second metformin is taking at approximately 1.00pm, just after a second glucose measurement. Activity levels are moderate during the day. The subject takes a third glucose measurement around 6pm, goes to sleep around 10.00pm, but wakes before midnight.

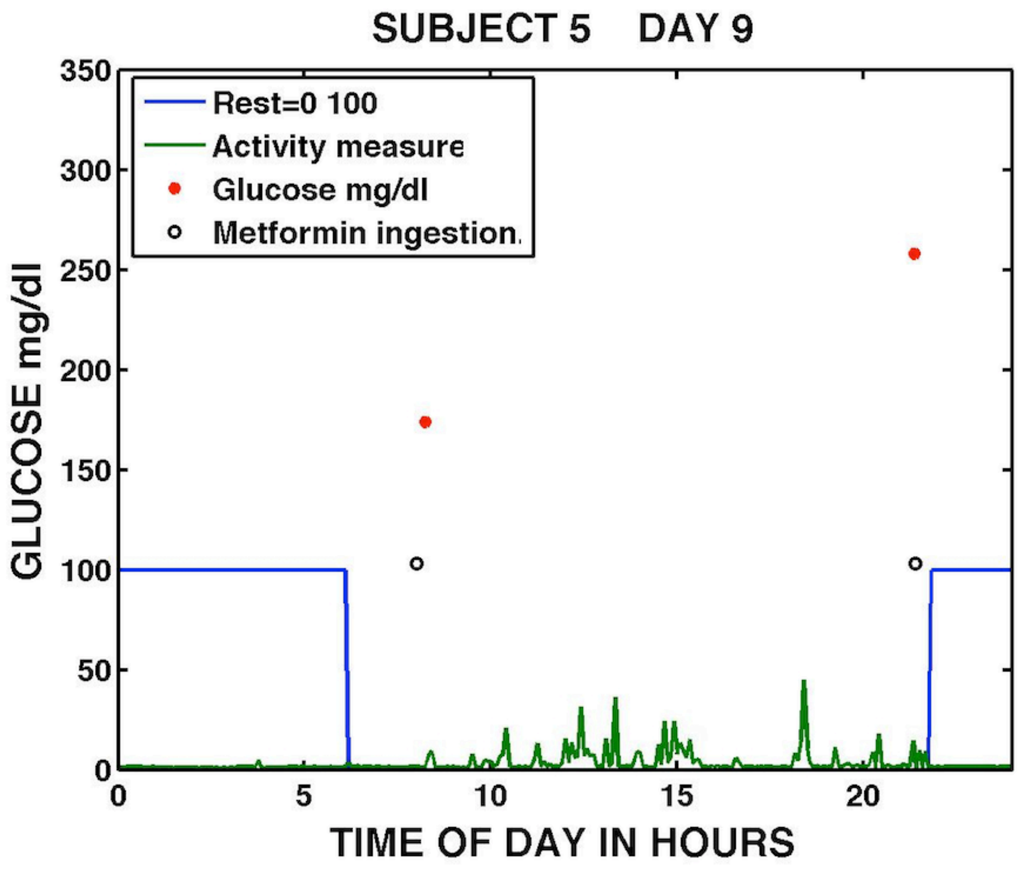

Graph 6: Daily medication taking, sleep/rest, activity, and glucose measu rement for Subject 5, Day 9. Graph shows continuous block of sleep until approximately 6.00am metformin ingestion and glucose measurement ar ound 8.00am, low grade activity during the day, metformin ingestion a nd glucose measurement just prior to sleep around 10.00pm.

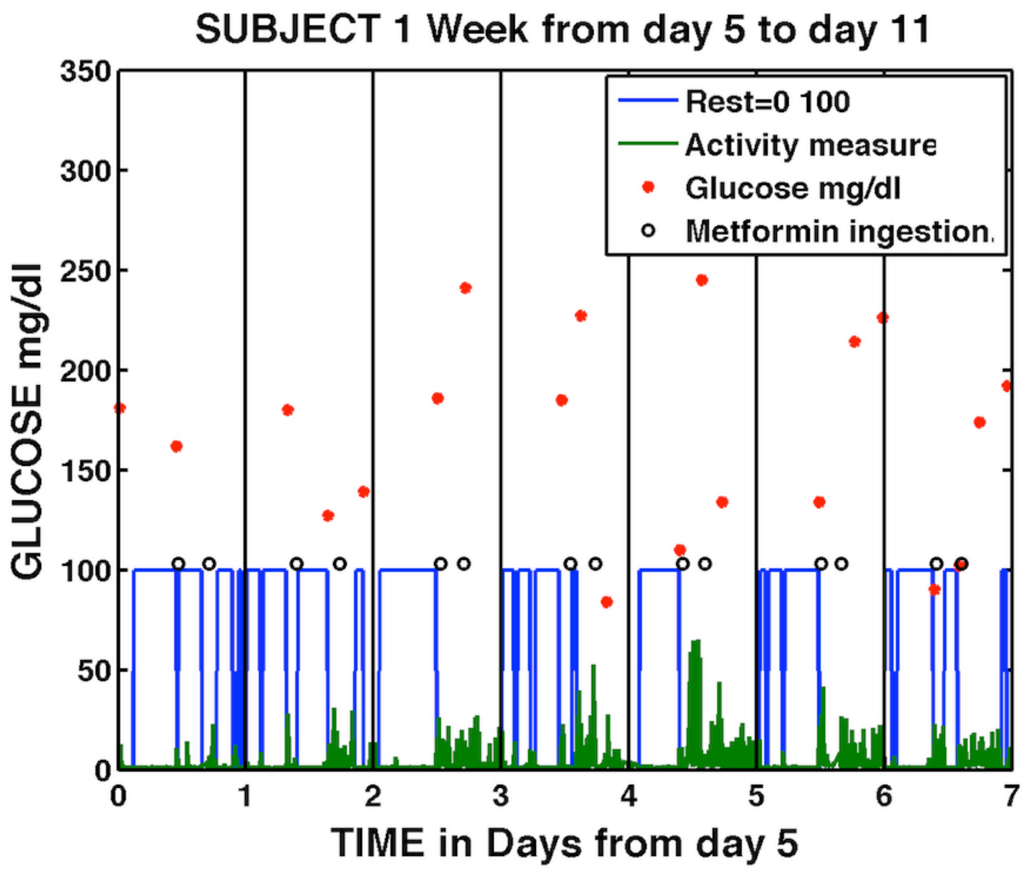

Graph 7: Weekly medication taking, sleep/rest, activity, and glucose measurement for Subject 1, Days 5-11.

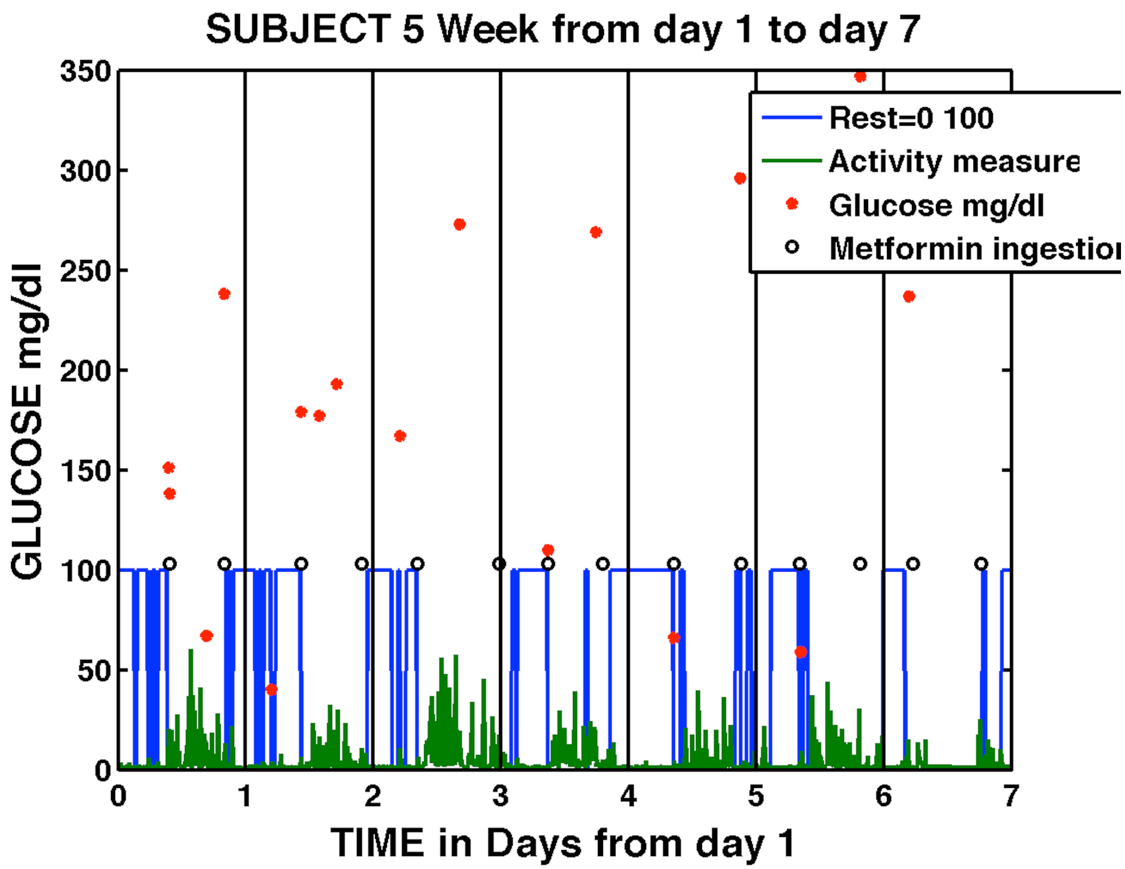

Graph 8: Weekly medication taking, sleep/rest, activity, and glucose measurement for Subject 5, Days 1-7.
